# MR imaging of fetal cardiac malposition and congenital cardiovascular anomalies on the four-chamber view

**DOI:** 10.1186/s40064-016-2833-x

**Published:** 2016-07-29

**Authors:** Su-Zhen Dong, Ming Zhu

**Affiliations:** Department of Radiology, Shanghai Children’s Medical Center, Shanghai Jiaotong University School of Medicine, 1678 Dongfang Rd., Shanghai, 200127 China

**Keywords:** Fetal heart, Congenital heart disease, Fetal echocardiography, Cardiac magnetic resonance imaging, Prenatal diagnosis

## Abstract

Fetal echocardiography is the method of choice to visualize the fetal congenital cardiovascular anomalies. However, there are some disadvantages. Fetal cardiac magnetic resonance imaging (MRI) has the potential to complement ultrasound in detecting congenital cardiovascular anomalies. This pictorial review draws on our experience about fetal cardiac MRI; it describes the four-chamber view on fetal cardiac MRI and important clues on an abnormal four-chamber view to the diagnosis of fetal congenital cardiovascular anomalies.

## Background

Abnormalities of the cardiovascular system are the most common congenital diseases in the fetus and the first cause of infant mortality (Johnson et al. 2014). Without a doubt, Echocardiography is the method of choice to visualize the fetal cardiac cardiovascular abnormalities.

Unlike fetal echocardiography imaging, cardiovascular magnetic resonance (CMR) is relatively unaffected by maternal and fetal conditions such as maternal obesity, uterine myoma, twins, oligohydramnios, fetal position and rib calcification, which particularly impair sonographic visualization of the fetal heart (Donofrio et al. 2014; Wielandner et al. 2013). Fetal CMR imaging has the potential to complement ultrasound in detecting cardiovascular malformations and extracardiac anomalies (Donofrio et al. 2014; Wielandner et al. 2013; Manganaro et al. 2014; Dong et al. 2013).

**Fig. 1:**
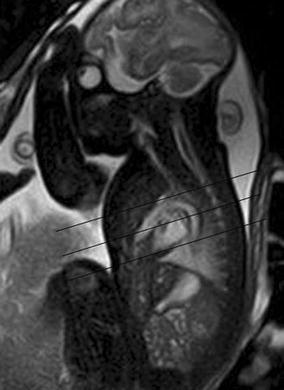
A 30-week fetus with the scan plane of four-chamber view. Fetal CMR B-TFE sagittal view image shows the scan plane of four-chamber view (scan *line*)

The four-chamber view is widely used as a main screening method for fetal cardiac defects (Dong and Zhu 2015). The four-chamber view of fetal CMR is similar but not absolutely same with the traditional four-chamber view of fetal echocardiography. The scan plane of four-chamber view is about 10°–15° caudal to cranial according to sagittal view imaging (Fig. 1). The four-chamber view of fetal CMR assesses cardiovascular anomalies by abnormal position, size, septum integrity, tumor and other malformations of the fetal heart. This pictorial review illustrates fetal cardiac malposition and congenital cardiovascular anomalies seen on the four-chamber view CMR imaging. The anomalies shown were confirmed by postnatal imaging and/or operation in all cases. The ethics committee of Shanghai Children’s Medical Center approved this study. All mothers gave written informed consent.

## Cardiac positional anomalies

**Fig. 2:**
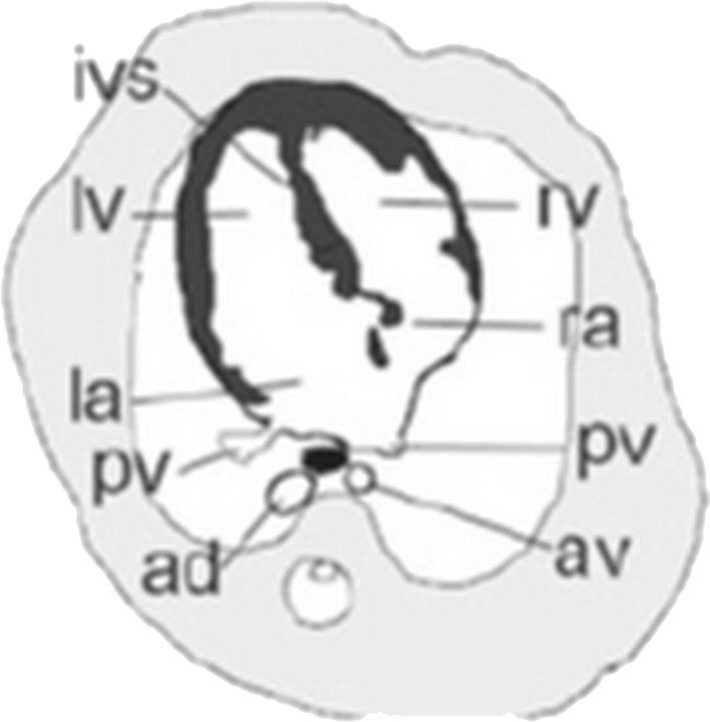
The scheme of figure of fetal four-chamber view. *ad* descending aorta, *av* azygos vein, *ivs* interventricular septum, *la* left atrium, *lv* left ventricle, *pv* pulmonary vein, *ra* right atrium, *rv* right ventricle

**Fig. 3:**
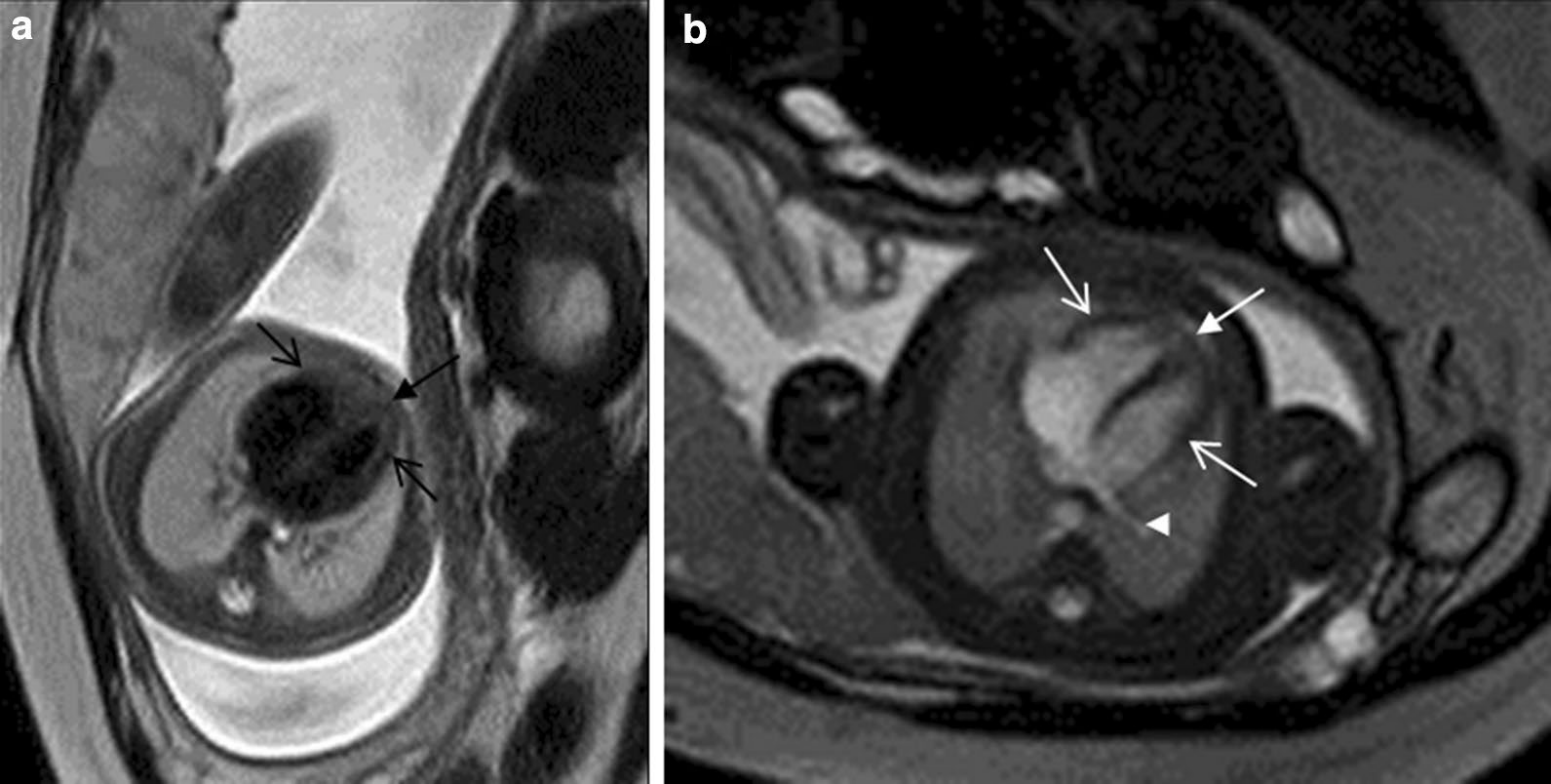
A 27-week fetus (SSTSE) and A 25-week fetus (B-TFE) with normal four-chamber view. Fetal CMR SSTSE and B-TFE four-chamber view images show the normal cardiac position (*arrow* in **a** and **b**), two ventricles (*open arrows* in **a** and **b**) and the pulmonary vein (*arrowhead* in **b**)

**Fig. 4:**
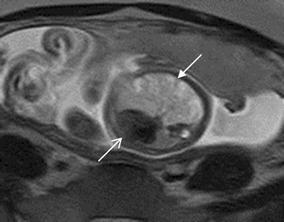
A 21-week fetus with CCAM. Fetal CMR SSTSE four-chamber view image shows that congenital cystic adenomatoid malformation in left lung (*arrow*) pushes the heart to the right side (*open arrow*)

**Fig. 5:**
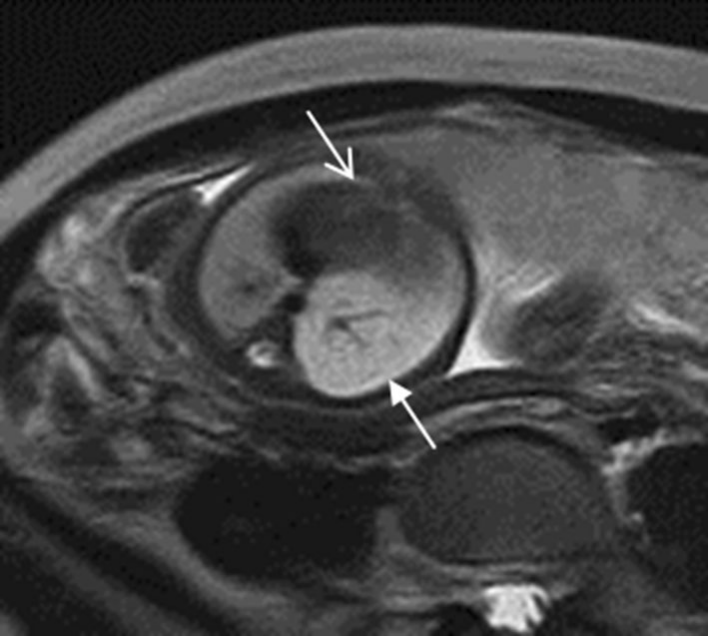
A 24-week fetus with BPS. Fetal CMR SSTSE four-chamber view image shows that bronchopulmonary sequestration in left lung (*arrow*) pushes the heart to the right side (*open arrow*)

**Fig. 6:**
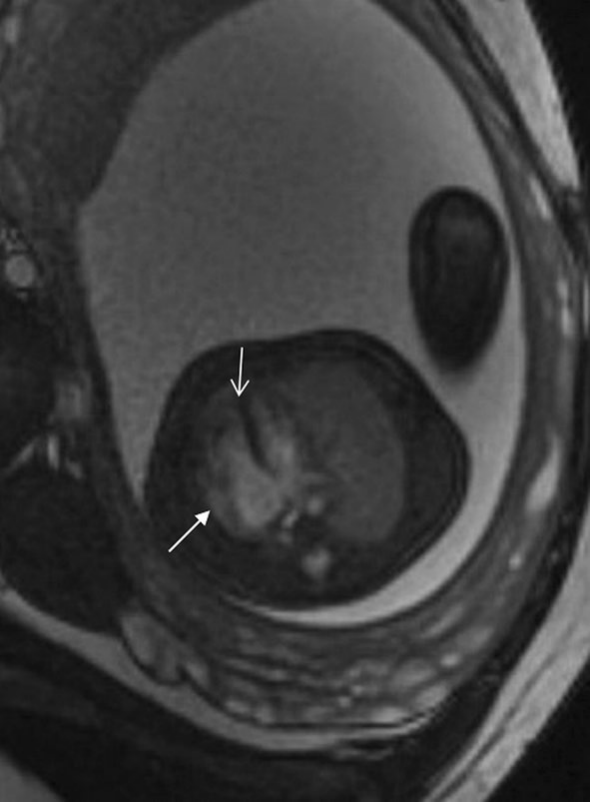
A 27-week fetus with the right pulmonary agenesis. Fetal CMR fast imaging employing steady-state acquisition (FIESTA, SSFP of GE) four-chamber view image shows the right pulmonary agenesis (*arrow*) and the heart shifting to the right side (*open arrow*)

**Fig. 7:**
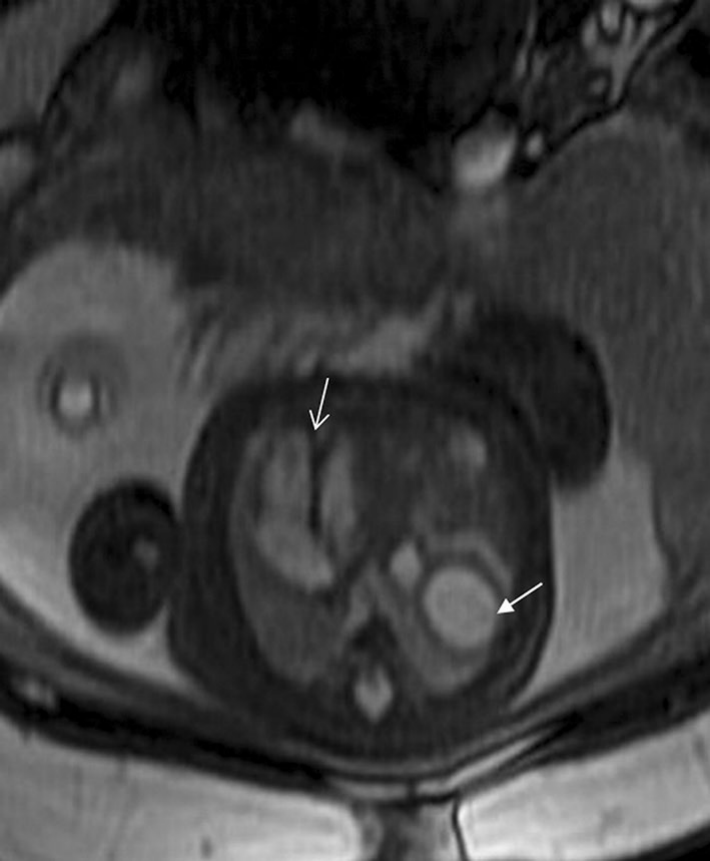
A 25-week fetus with the left congenital diaphragmatic hernia. Fetal CMR FIESTA four-chamber view image shows the stomach herniated into the left chest (*arrow*) and the heart pushed to the right side (*open arrow*)

**Fig. 8:**
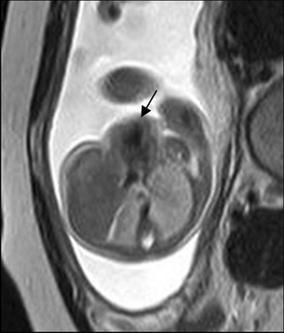
A 25-week fetus with pentalogy of Cantrell syndrome. Fetal CMR SSTSE four-chamber view image shows the heart protruding out of the sternal defect (*arrow*)

**Fig. 9:**
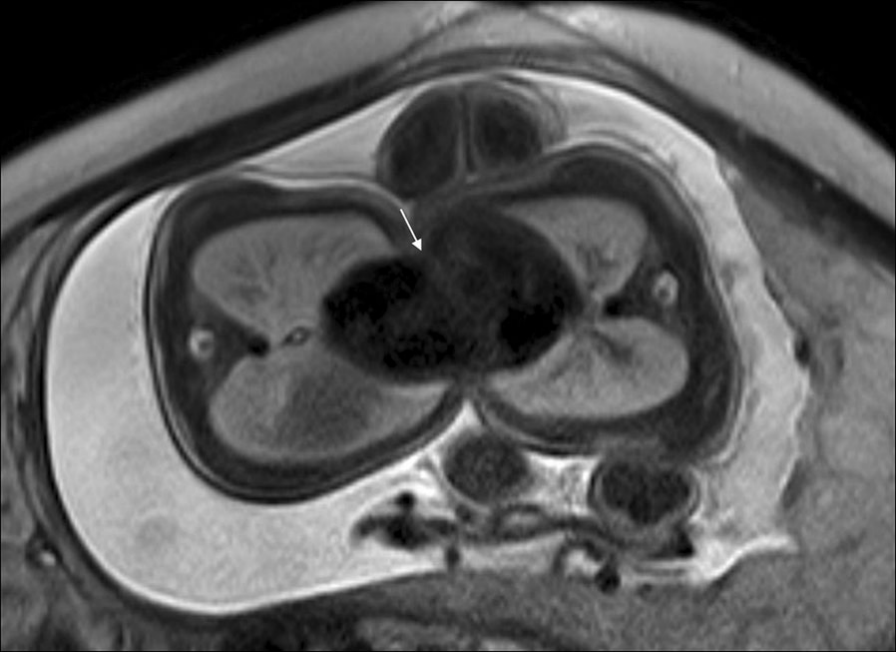
A 26-week fetus with thoracopagus conjoined twins. Fetal CMR SSTSE four-chamber view image shows the two adjacent unshared fetal heart and shared pericardium (*arrow*)

**Fig. 10:**
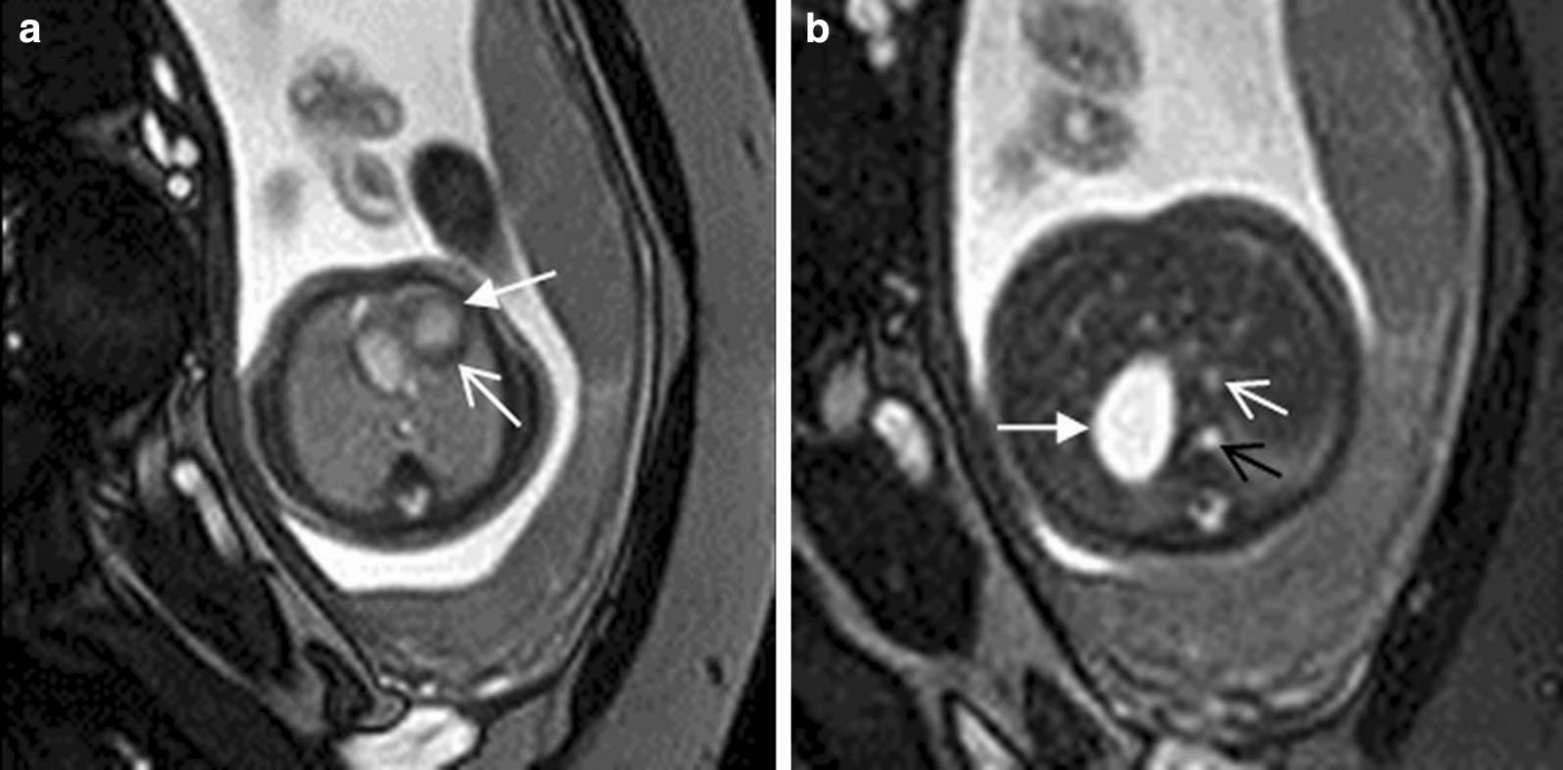
A 26-week fetus with asplenia syndrome. Fetal CMR B-TFE four-chamber view image shows levocardia (*arrow* in **a**), right-sided stomach (*arrow* in **b**), single ventricle (*open arrow* in **a**) and both left descending aorta (*black open arrow* in **b**) and inferior vena cava (*white open arrow* in **b**)

The anatomy of fetal heart can be clearly demonstrated on four-chamber view (Fig. 2; Brugger 2010). Fetal cardiac positional anomalies are rare but very important. The cardiac axis can be clearly demonstrated on four-chamber view. While positional anomalies of fetal heart may also be seen on single-shot turbo spin-echo (SSTSE) sequences (Fig. 3a), they can be better assessed on steady-state free precession (SSFP) sequences (Fig. 3b). For every examination, the initial assessment must include determination of fetal cardiac position and accurate assessment of visceral and atrial situs (Falkensammer et al. 2008). Positional anomalies may be an isolated finding, such as simple dextrocardia and mesocardia, or may be caused by the mass effect of extracardiac anomalies (Recio Rodríguez et al. 2012), such as congenital cystic adenomatoid malformation (CCAM) of lung (Fig. 4), bronchopulmonary sequestration (BPS) (Fig. 5), pulmonary hypoplasia (Fig. 6) and congenital diaphragmatic hernia (Fig. 7). However, they may also be part of more complex malformations, such as ectopia cordis (Fig. 8), conjoined twins (Fig. 9) or heterotaxy syndromes (Fig. 10a, b). The four-chamber view can simultaneously show abnormal cardiac axis caused by extracardiac thoracic masses or due the presence of heterotaxy syndrome and extracardiac thoracic masses due to a large field of view in MR imaging (Figs. 4, 5, 6, and 7).

## Cardiac and ventricle size anomalies

Fetal cardiac size anomalies also are very important and should be secondly assessed. The cardiac size relative to the thorax may be evaluated from four-chamber view CMR images. Cardiac malformations associated with cardiomegaly and/or different sizes of the cardiac chambers are also easily recognized on four-chamber view of fetal CMR.

**Fig. 11:**
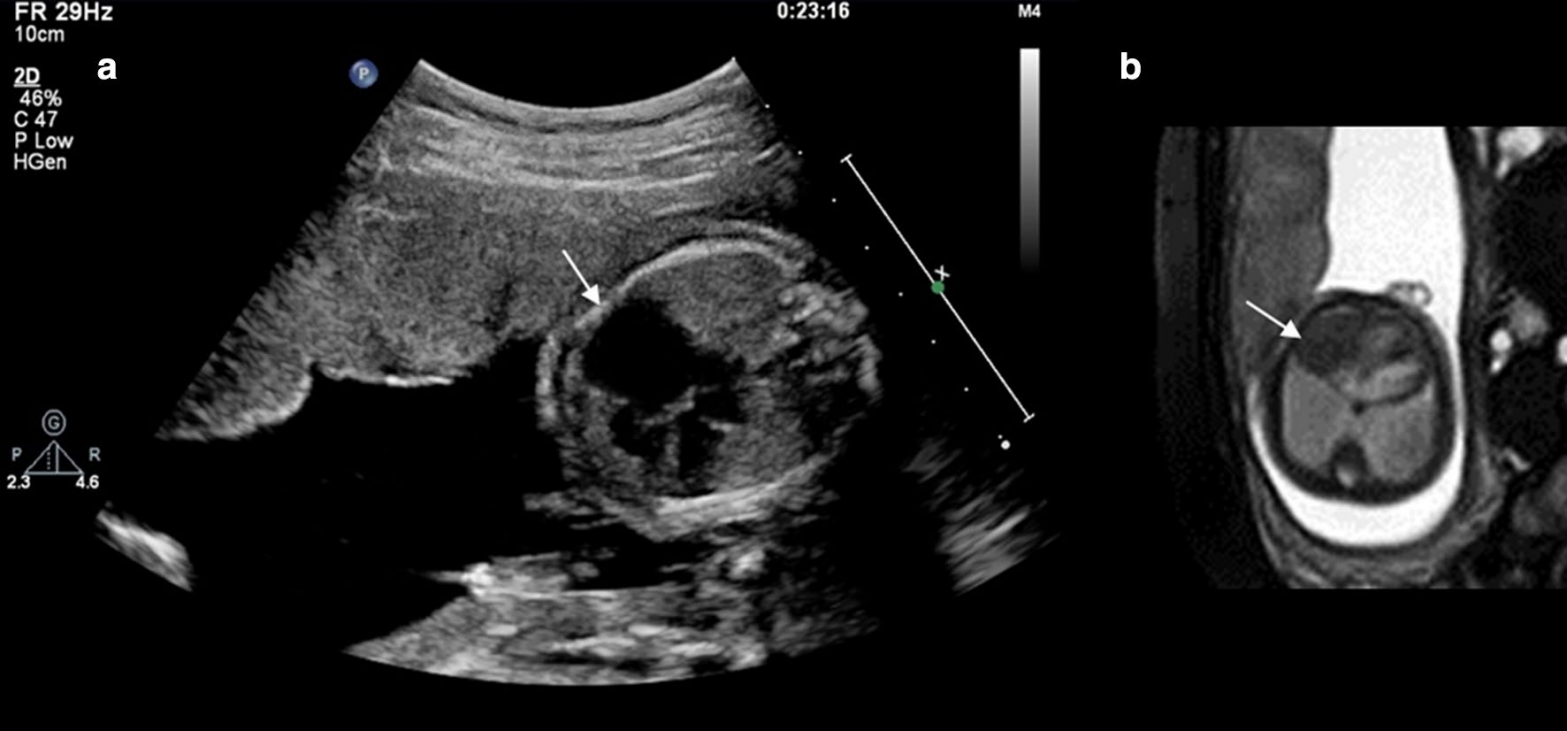
A 25-week fetus with Ebstein anomaly. Fetal echocardiography and CMR B-TFE four-chamber view image shows the flow regurgitation is from right ventricle to right atrium and significantly dilated right atrium (*arrows* in **a** and **b**)

**Fig. 12:**
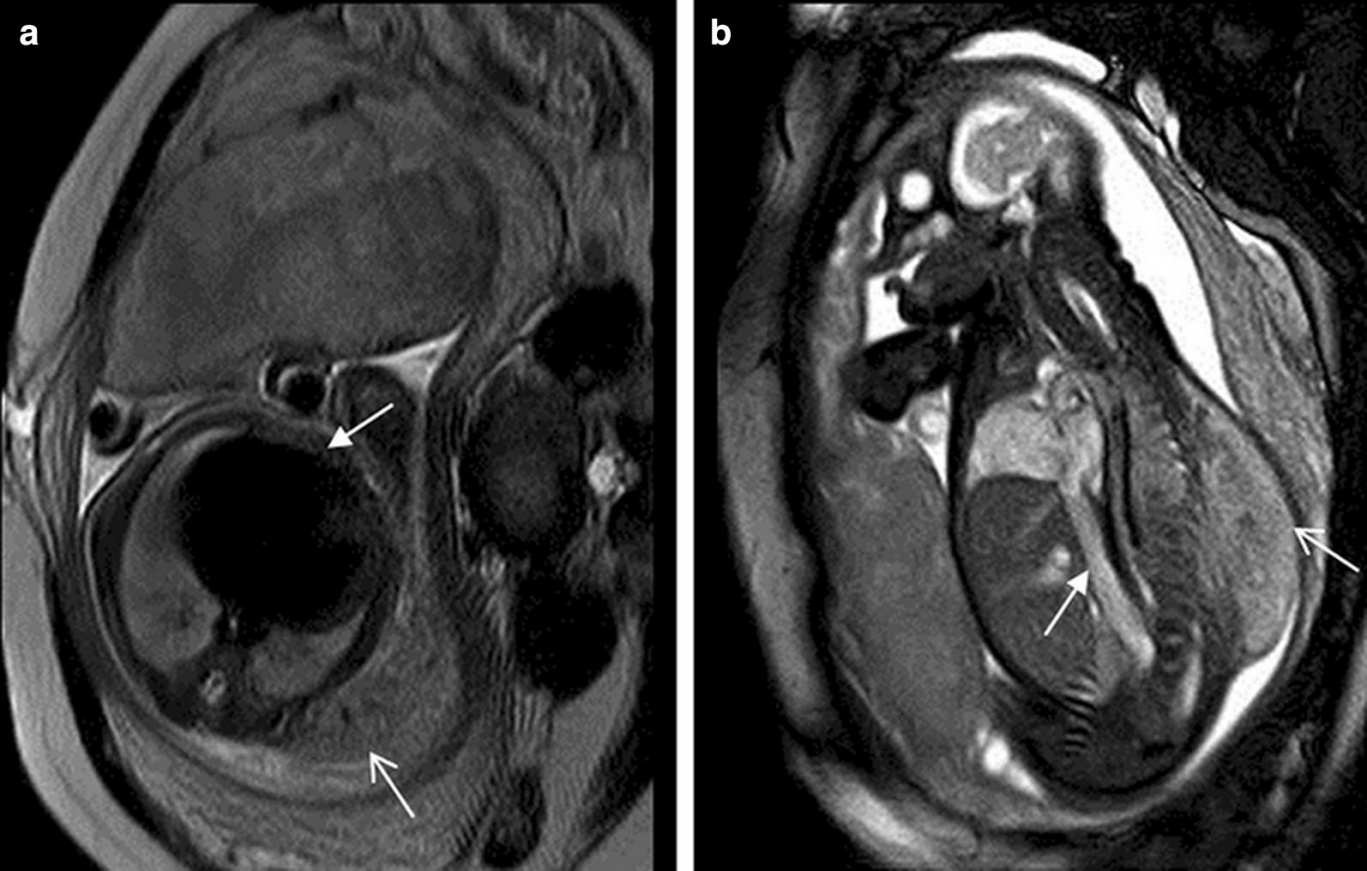
A 34-week fetus with the large hemangioendothelioma. Fetal CMR SSTSE four-chamber and B-TFE sagittal view images show the cardiomegaly (*arrow* in **a**), the large hemangioendothelioma on the left chest wall (*open arrow* in **a**) and the back (*open arrow* in **b**) and dilated inferior vena (*arrow* in **b**)

Fetal cardiomegaly refers to an enlarged fetal heart. It can arise from a number of situations which include congenital cardiac anomalies such as Ebstein anomaly (Fig. 11a, b), twin–twin transfusion syndrome and abnormal shunting such as vein of Galen malformation and large hemangioendothelioma (Avni et al. 2009; Fig. 12a, b). The four-chamber view cine SSFP images allow the detection of the regurgitation across the atrioventricular valves due to cardiomegaly, which is more frequent at the tricuspid valve.

**Fig. 13:**
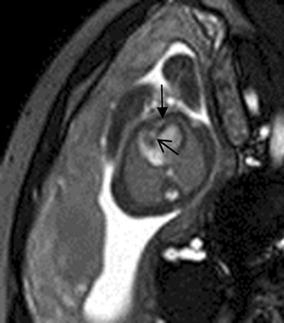
A 21-week fetus with tricuspid atresia. Fetal CMR B-TFE four-chamber view image shows the absence of the connection between the right atrium and the right ventricle, the hypoplastic right ventricle (*arrow*) with an associated ventricular septal defect (*open arrow*)

**Fig. 14:**
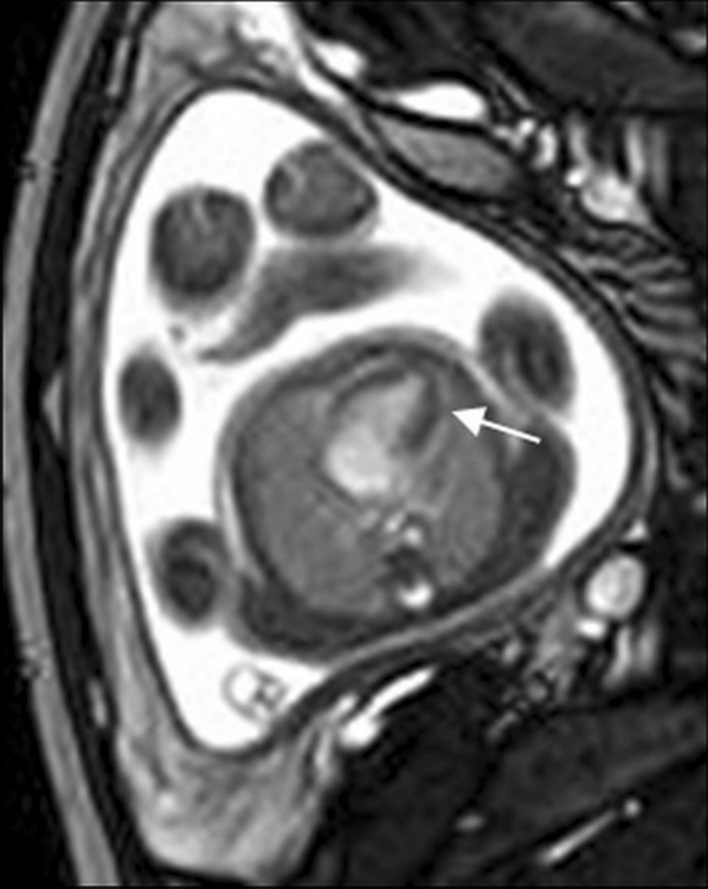
A 22-week fetus with hypoplastic left heart syndrome. Fetal CMR B-TFE four-chamber view image shows the tiny left ventricle (*arrow*)

**Fig. 15:**
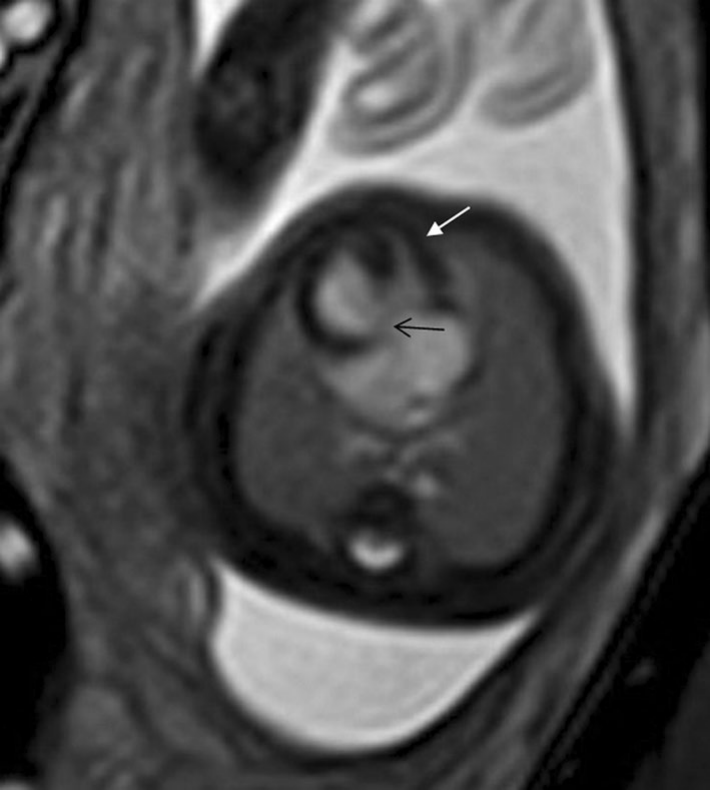
A 22-week fetus with asplenia syndrome. Fetal CMR B-TFE four-chamber view image shows the small left ventricle (*arrow*) and complete atrioventricular septal defect (*open arrow*)

The two atria and two ventricles are approximately equal in size, although the right side of the heart becomes slightly larger as gestation progresses. Cardiac malformations associated with different sizes of the cardiac chambers, such as tricuspid atresia (Fig. 13), hypoplastic left/right heart (Freud et al. 2014; Fig. 14) and univentricular heart (Figs. 10a, 15), are very important and are also easily recognized with four-chamber view CMR images. In pulmonary atresia with intact ventricular septum, the size of the right ventricle may show a wide range, from hypoplasia to dilatation. If fetus has only one ventricle large enough to pump effectively, a bi-ventricular repair is impossible. The Fontan procedure will be used in patient with single functional ventricle. The Fontan procedure is a palliative surgical procedure. The four-chamber view of SSFP sequences can more visually display cardiac and ventricle size because of the high blood signal compared to ultrasound.

## Cardiac septum defects

**Fig. 16:**
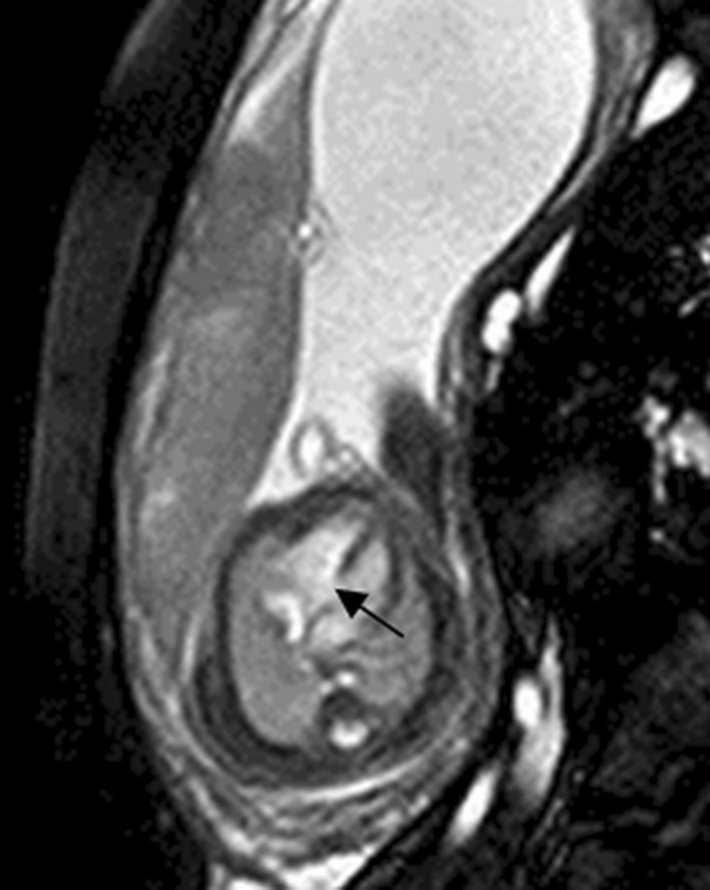
A 26-week fetus with ventricular septal defect. Fetal CMR B-TFE four-chamber view image shows the perimembranous ventricular septal defect (*arrow*)

**Fig. 17:**
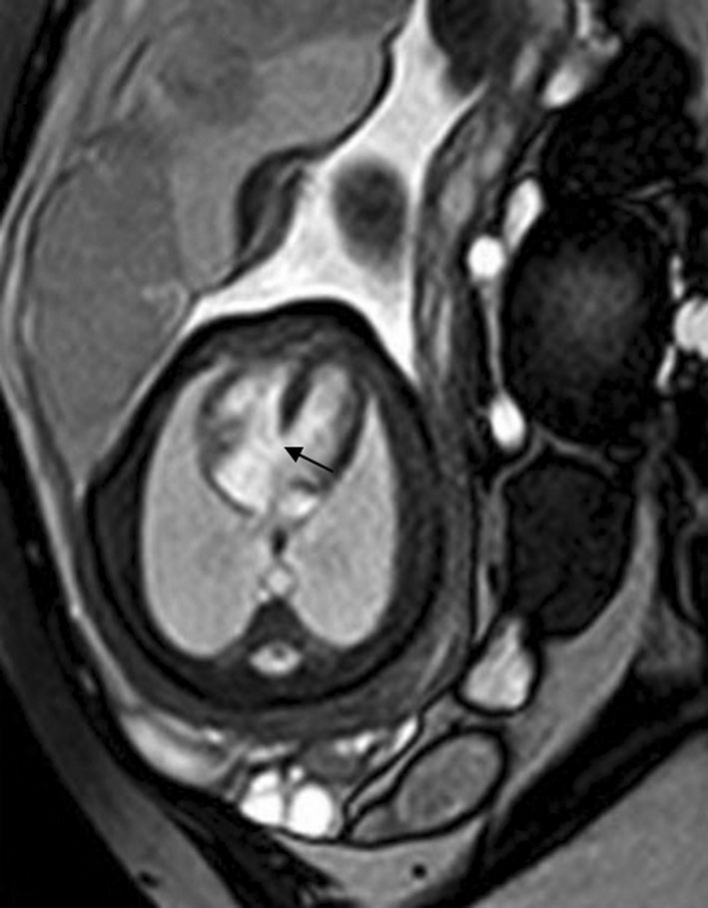
A 28-week fetus with complete atrioventricular septal defect. Fetal CMR B-TFE four-chamber view image shows the defects in the atrial and ventricular septum at the level of the atrioventricular connections (*arrow*)

Fetal cardiac septum defects are common and important. The crux of the heart, the area of junction of atrial septum, atrioventricular valves, and interventricular septum should be intact and the ventricular septum should be intact on from four-chamber view CMR images. The limitation of fetal CMR is the difficulty in detecting small ventricular septum defects (Manganaro et al. 2014; Dong et al. 2013). The large perimembranous ventricular septum defect (Fig. 16) and the ventricular septum defect located in the muscular part of the septum may be relatively easy to be found with four-chamber view on fetal CMR. The four-chamber view CMR image also allows the detection of atrioventricular septal defect (Craig 2006). In a complete atrioventricular septal defect, there is a combination of defects in the atrial septum primum and ventricular septum at the level of the atrioventricular connections (Figs. 15, 17). Fetal CMR usually don’t diagnose secundum atrial septal defect. The four-chamber view CMR image allows the detection of the foramen ovale. Fetuses with critical congenital heart disease such as hypoplastic left heart syndrome, complete transposition of great arteries and severe right heart obstruction are dependent on the foramen ovale patency (Harold 2014). The four-chamber view can additionally display cardiac septum defects when echocardiography has limitations.

## Cardiac tumor and other malformations

**Fig. 18:**
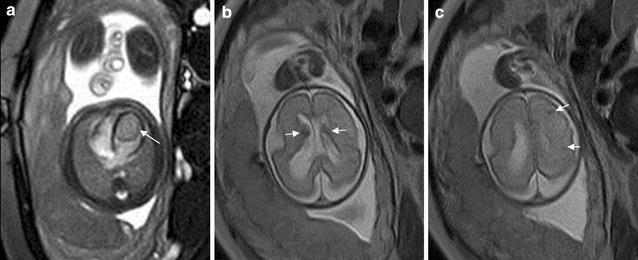
A 26-week fetus with tuberous sclerosis. Fetal CMR B-TFE four-chamber and head SSTSE axial view images show the large intracardiac rhabdomyomas in left ventricle (*arrow* in **a**), small subependymal (*arrows* in **b**) and cortical tubers (*arrows* in **c**)

Cardiac rhabdomyoma is the most common primary fetal tumor of the heart. They can be single or multiple. Although the behavior of a cardiac rhabdomyoma is benign and can be spontaneous regression, the positioning within critical areas in the heart can lead to chamber obstruction. The presence of cardiac rhabdomyomas is strongly suggestive of tuberous sclerosis (Atalay et al. 2010). On four-chamber view SSFP image, rhabdomyomas have intermediate signal intensity (Fig. 18a). MRI is also used to evaluate the presence of tuberous sclerosis involving the brain compared to ultrasound (Fig. 18b, c), kidneys, and liver (Goel et al. 2016).

**Fig. 19:**
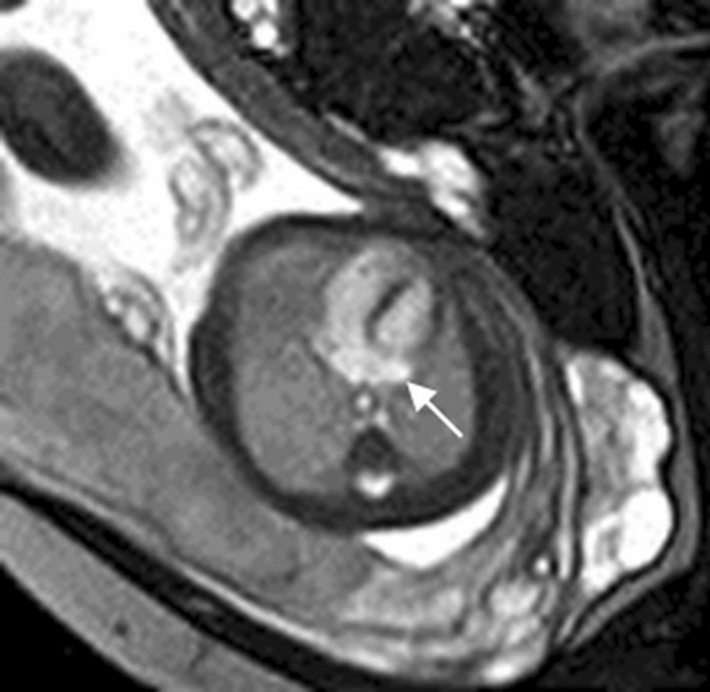
A 27-week fetus with persistence of the persistent left superior vena cava. Fetal CMR B-TFE four-chamber view image shows an enlarged coronary sinus (*arrow*)

**Fig. 20:**
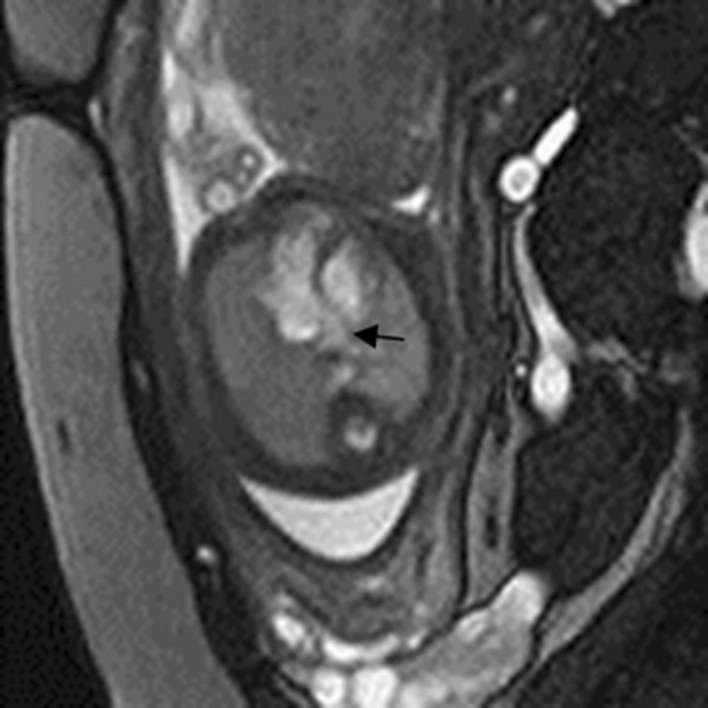
A 26-week fetus with total anomalous pulmonary venous connection. Fetal CMR B-TFE four-chamber view image shows the pulmonary veins draining into a large coronary sinus (*arrow*)

**Fig. 21:**
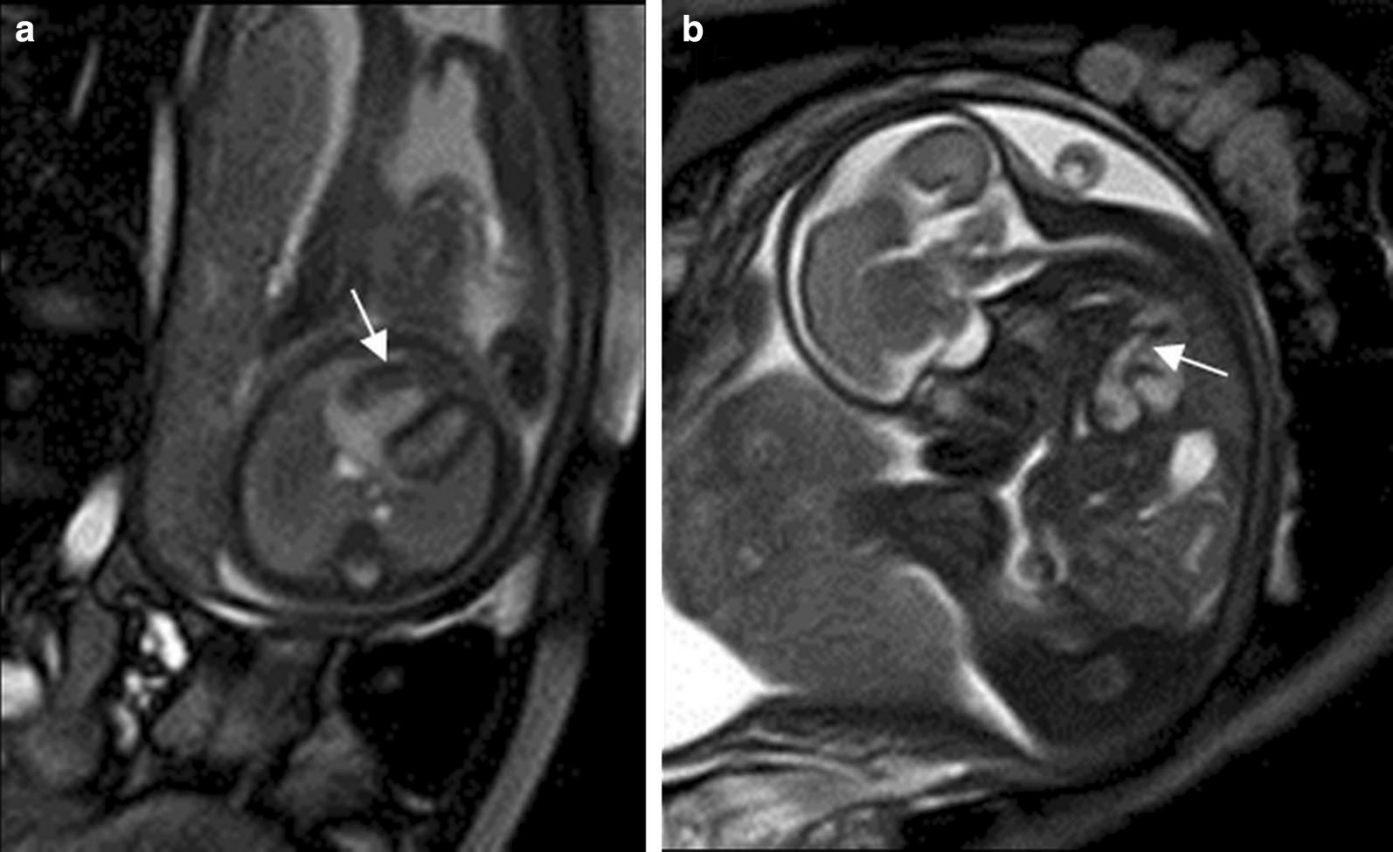
A 26-week fetus with pulmonary valve stenosis. Fetal CMR B-TFE four-chamber and sagittal view images show the right ventricle hypertrophy (*arrow* in **a**) and the jet sign (*arrow* in **b**)

**Fig. 22:**
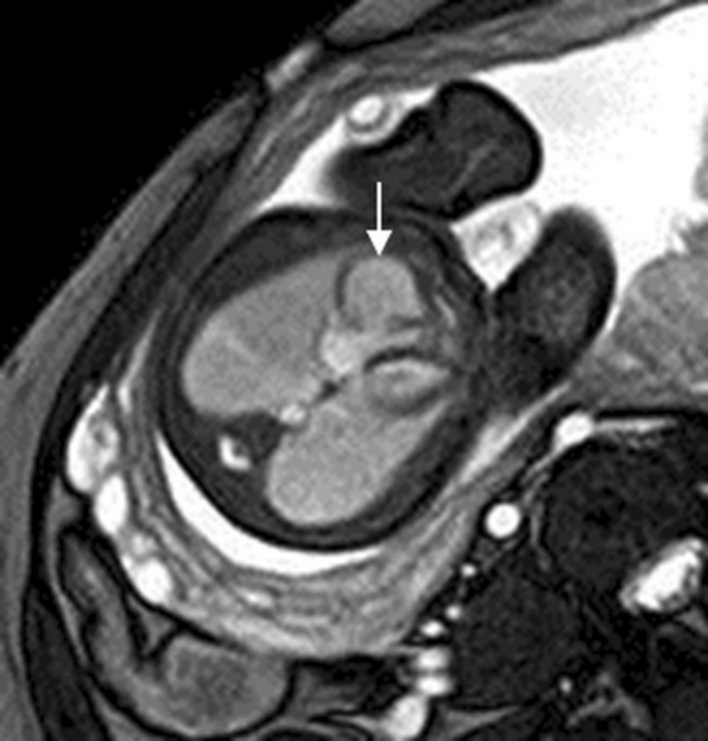
A 31-week fetus with right ventricle diverticulum. Fetal CMR B-TFE four-chamber view image shows the large right ventricle diverticulum (*arrow*)

An evaluation of four-chamber view SSFP image should also provide some information of systemic (Fig. 19) and pulmonary venous connections (Fig. 20), atrial and ventricular connections (Manganaro et al. 2009), ventricular wall thickness (Fig. 21), heart diverticulum (Fig. 22), pericardium cyst, pericardium effusion and others. SSTSE sequences were more valuable in the identification of pericardium cyst and pericardium effusion compared to ultrasound, due to cyst and fluid with high signal, however, cardiac chambers with low signal.

## Conclusion

The four-chamber view images on fetal CMR can identify cardiac malposition, cardiac malformations associated with cardiomegaly, different sizes of the cardiac chambers, large cardiac septum defects, cardiac tumors and others. Although certain fetal heart abnormalities will not be consistently identified such as small ventricular septum defect and valvular stenosis, the four-chamber view on fetal CMR still can provide some diagnostic information for fetal heart anomalies.
